# The Assessment on Synergistic Activity of Ebselen and Silver Ion Against *Yersinia pseudotuberculosis*

**DOI:** 10.3389/fmicb.2022.963901

**Published:** 2022-07-25

**Authors:** Chuanjiang Dong, Wei Chen, Lili Zou, Binbin Liu, Kaihong Deng, Dingrui Guo, Peng Wang, Hao Chen, Helen Wang, Jun Wang

**Affiliations:** ^1^The First College of Clinical Medical Science, China Three Gorges University, Yichang, China; ^2^Hubei Key Laboratory of Tumor Microenvironment and Immunotherapy, Medical College, China Three Gorges University, Yichang, China; ^3^The Institute of Infection and Inflammation, Medical College, China Three Gorges University, Yichang, China; ^4^Affiliated Second People’s Hospital of China Three Gorges University, Yichang, China; ^5^Department of Medical Biochemistry and Microbiology, Uppsala University, Uppsala, Sweden; ^6^The People’s Hospital of China Three Gorges University, Yichang, China

**Keywords:** *Yersinia pseudotuberculosis*, Ebselen, silver nitrate, antibacterial activity, immune-modulatory property

## Abstract

*Yersinia pseudotuberculosis* is a foodborne zoonotic bacterium that is pathogenic to guinea pigs, rabbits, and mice. It also causes pseudotuberculosis in humans. However, it still lacked the scientific basis for control. Here, we found out that Ebselen (EbSe) exhibited synergistic antibacterial activity with silver nitrate (Ag^+^) against *Y. pseudotuberculosis* YpIII strain with high efficacy *in vitro* using UV-visible light absorption spectrum, 5,5’-dithiobis-(2-nitrobenzoic acid), laser scanning confocal microscope, flow cytometry, transmission electron microscopy and Western blotting assays. The depletion of total glutathione (GSH) amount and inhibition of thioredoxin reductase (TrxR) activity in thiol-dependent redox system revealed the destructiveness of EbSe-Ag^+^-caused intracellular oxidative stress. Furthermore, a YpIII-caused mice gastroenteritis model was constructed. EbSe-Ag^+^ significantly reduced bacterial loads with low toxicity. It also down-regulated the expression levels of interferon (IL)-1β and tumor necrosis factor-α, up-regulated the expression level of IL-10 on-site. All the *in vivo* results demonstrated the antibacterial activity and immune-modulatory property of EbSe-Ag^+^. Collectively, these results provided academic fundament for further analysis and development of EbSe-Ag^+^ as the antibacterial agents for pseudotuberculosis control.

## Introduction

Yersiniosis, one of the “other infectious diarrheas” in humans, is an important foodborne zoonosis with wide range of clinical symptoms caused by the enteric pathogens *Yersinia enterocolitica* and *Yersinia pseudotuberculosis*, which is the third most common bacterial enteritis disease in European countries (Mecsas, 2019). Although human *Y. pseudotuberculosis* infections are less frequent than those caused by *Y. enterocolitica* worldwide, it has a wide animal reservoir, mostly in temperate and cold countries (Voskresenskaya et al., 2014). Pseudotuberculosis in animals can lead to tuberculosis-like symptoms, including localized tissue necrosis and granulomas in the liver, spleen, and lymph nodes (Aswal et al., 2020). *Y. pseudotuberculosis* infections in humans are acquired through the gastrointestinal tract by the ingestion of contaminated food products and the clinical manifestations of pseudotuberculosis are diarrhea, abdominal pain, and fever (Ohnishi et al., 2021; Rivas et al., 2021). The frequency of human infection caused by certain yersinia subgroups might be related to the frequency of exposure to specific animal sources (Le Guern et al., 2016). In some specific areas, *Y. pseudotuberculosis* may also cause a specific disease known as Far East scarlet-like fever for its clinical similarities to scarlet fever caused by group A streptococci (Dakić et al., 2005; Amphlett, 2016). Such atypical infections are severe, characterized by a strong inflammatory syndrome accompanying intestinal disorders (Eppinger et al., 2007).

As a “generalist,” *Y. pseudotuberculosis* deploys sophisticated virulence factors to effectively evade the host immune system (Bergsbaken et al., 2009; Lima-Junior et al., 2013; Reinhardt et al., 2018; Schneiders et al., 2021), and host factors are also essential for pathogen-induced cell death (Mares et al., 2021). Hypoxic environment of the intestine is critical for nutrient absorption, intestinal barrier function, and innate and adaptive immune responses in the intestine (Singhal and Shah, 2020). The oxygen deficiency during growth promotes increasing of pathogenic potential of *Y. pseudotuberculosis* (Bakholdina et al., 2009), the outcomes of artificial oxidative stress arouse our curiosity.

Known as thiol dependent redox system (TDRS), thioredoxin (Trx) and glutathione (GSH) systems are vital defenders against oxidative stress (Ren et al., 2020). Trx system consists of Trx and thioredoxin reductase (TrxR); The GSH system be formed from GSH, glutathione reductase (GR) and glutaredoxin (Grx). Both systems work as backup to maintain the intracellular redox homeostasis, participating in signal transduction and regulation, DNA repair and protein synthesis and repair, oxidation resistance and other biological processes. Ultimately, TDRS affects bacterial survival and death, activation, and proliferation (Ren et al., 2018, 2020).

Our previous work showed that a non-toxic seleno-organic drug, Ebselen (EbSe), has antibacterial effect against Gram-positive bacteria (Lu et al., 2013; Dong et al., 2019); meanwhile, it could work synergistically with silver nitrate (Ag^+^) to kill a couple of Gram-negative bacteria such as multidrug-resistant *Escherichia coli* (Zou et al., 2017) and *Acinetobacter baumannii* (Dong et al., 2020) by disrupting the TDRS and promoting the oxidative stress in bacteria (Zou et al., 2018; Dong et al., 2019). Thus, the *in vitro* and *in vivo* outcomes of EbSe-Ag^+^ against *Y. pseudotuberculosis* YpIII strain have been extensively investigated to develop its scientific basis for pseudotuberculosis control.

## Materials and Methods

### Mouse and Bacterial Strains

Kunming mice (male, 22–25 g) were purchased from China Three Gorges University, and the ethical permit approval of the Medical Animal Care and Welfare Committee of China Three Gorges University was obtained before using the mice for study. Every five mice were kept in an individual cage with a constant dark-light cycle in a conventional SPF animal house and were given free access to fundamental food as normal diet and water.

*Yersinia pseudotuberculosis* YpIII strains with different reporter systems on the virulence plasmid were constructed as described previously (Wang et al., 2016) and listed in Table 1. All experiments were carried out in the BSL2 laboratory.

**TABLE 1:** YpIII strains with different reporter systems.

Strain	Genotype	Experiments	Source
YpIII-GFP	YPIII (pCD1 expressing GFP, Km^r^)	UV-Vis; LS-MS PAE; TEM DTNB; WB	This study
YpIII-mCherry	YPIII, (pCD1 expressing mCherry, Km^r^)	UV-Vis; PAE DTNB; FCM WB	This study
YpIII-bioluminescent	YPIII, Xen4 (pCD1 with Tn1000:Tn5 luxCDABE, Km^r^)	Animal experiments	Dong et al., 2020

### Reagents

Luria Bertani (LB) medium (EMD Millipore), 2-phenyl-1, 2-benzisoselenazol-3(2H)-one (EbSe) (Daiichi), protease inhibitor cocktails (Roche), anti-Trx1 polyclonal antiserum (IMCO), rabbit anti-sheep IgG-HRP (Santa Cruz), goat anti-mouse H&L antibodies (Santa Cruz), IgG2a mouse monoclonal antibody (VIROGEN), CellROX™ Deep Red Reagent (Invitrogen), Anti-IL-1β antibody (Proteintech), anti-TNF-α antibody (Proteintech), anti-IL-10 antibody (Proteintech). Protein inhibitor cocktail (MedChemExpress). Silver nitrate and all the other reagents were from Sigma-Aldrich.

### Inhibitory Effect of EbSe-Ag^+^ Against YpIII

The inhibitory effect of EbSe-Ag^+^ on the growth of YpIII was measured by UV-Vis at A_600_. YpIII cells were grown (26°C, 200 rpm) 8 h and diluted 1,000 times and treated with a serial concentration of EbSe-Ag^+^ for 24 h at 26°C in a 96-well plate, and the A_600_ were measured.

### Bactericidal Effect of EbSe-Ag^+^ Against YpIII

The bactericidal activity of EbSe-Ag^+^ against YpIII was detected by laser scanning confocal microscopy (LSCM, A1R^+^, Nikon). YpIII cells were grown (26°C, 200 rpm) until an A_600_ of 0.4, and diluted 1,000 times, which were treated with 4 μM EbSe and 0.5 μM Ag^+^ for 24 h at 26°C. The bactericidal effect was observed by LSCM.

### EbSe-Ag^+^ on YpIII Bacterial Morphology

YpIII was grown until an A_600_ of 0.4 and treated for 30 min with 80 μM EbSe and 5 μM Ag^+^. Cells were obtained by centrifugation (4°C, 12,000 rpm, 15 min) and fixed with 2.5% glutaraldehyde. The morphology of YpIII cells was observed by transmission electron microscopy (TEM, Hitachi H-7500).

### Post-antibiotic Effect of EbSe-Ag^+^ Against YpIII

The post-antibiotic effect (PAE) of EbSe-Ag^+^ against YpIII was detected by UV-Vis. YpIII cells were grown (26°C, 200 rpm) until an A_600_ of 0.4, and diluted 1,000 times. Further, cells were treated with 4 μM EbSe and 0.5 μM Ag^+^ for 32 h at 26°C, 200 rpm. Then, drugs were removed by centrifugation (4°C, 10,000 rpm, 2 min) for three times using PBS (pH 7.4). The A_600_ of YpIII cells were further measured every 1 h for 6 h.

### The Rescue Effect of DTT Against EbSe-Ag^+^-Treated YpIII

YpIII cells were grown (26°C, 200 rpm) until an A_600_ of 0.4, and diluted 1,000 times, which were treated with 4 μM EbSe and 0.5 μM Ag^+^ after pre-incubation with 4 μM dithiothreitol (DTT) for 30 min at 26°C, 200 rpm. The A_600_ of YpIII cells were measured.

### The Disruption of Thioredoxin Reductase Activity and Glutathione Amount by EbSe-Ag^+^

YpIII cells were cultured until an A_600_ of 0.4 and incubated with 80 μM EbSe and 5 μM Ag^+^ for 30 min. YpIII cells were obtained by centrifugation (4°C, 5,000 rpm, 5 min), and a protein inhibitor cocktail (in 50 mmol/L Tris-EDTA buffer, pH 7.4) was added to decrease the protease activity. The cells were disrupted with sonication (240 W, 10 min), and the supernatants were obtained by centrifugation (4°C, 12,000 rpm, 10 min). The TrxR activity and total glutathione amount were detected in a 96-well plate by 5, 5’-dithiobis-(2-nitrobenzoic acid) assay. The activity of the untreated group was 100%. The protocols were performed as previously described (Lu et al., 2013; Zou et al., 2017; Wang P. et al., 2020).

### Protein Expression Level of Thioredoxin 1 Upon Treatment With EbSe-Ag^+^

YpIII cells were cultured until an A_600_ of 0.4, which were incubated with 80 μM EbSe and 5 μM Ag^+^ for 30 min. After lysis by sonication, the cell lysates were obtained by centrifugation at 12,000 rpm for 20 min. Western blotting assay was performed with anti-Trx1 polyclonal antibody.

### Protein *S*-glutathionylation Level Upon Treatment With EbSe-Ag^+^

YpIII cells were cultured until an A_600_ of 0.4, which were incubated with 80 μM EbSe and 5 μM Ag^+^ for 30 min. After lysis by sonication, the cell lysates were obtained by centrifugation at 12,000 rpm for 20 min. Total protein *S*-glutathionylation (*S*-PSSG) of 80 μM EbSe and 5 μM Ag^+^-treated YpIII cells were detected by Western blotting. Cells were cultured, washed, and resuspended in buffer containing 30 mmol/L Iodoacetamide (IAM). Western blotting assay was performed with IgG2a mouse monoclonal antibody for glutathione-protein complexes.

### ROS Production by EbSe-Ag^+^ Treatment

YpIII were grown until an A_600_ of 0.4 in LB medium and incubated with 80 μM EbSe and 5 μM Ag^+^ for 30 min. The YpIII cells were stained with 5 μM CellROX™ Deep Red Reagent for 30 min at 37°C. After incubation, the reactive oxygen species (ROS) production was quantified by flow cytometry (BECKMAN COULTER, AW15093).

### Acute Mice Gastroenteritis Model

All experiments were performed in accordance with the relevant guidelines and regulations approved by China Three Gorges University. 60 healthy male Kunming mice were randomly divided into 5 groups randomly (*n* = 12). The mice were infected via intragastric gavage with 100 μL 2 × 10^9^CFU of YpIII as previously described (Wang et al., 2016) and were further administered i.p. with 20 mg/kg EbSe, 5 mg/kg Ag^+^, 20 mg/kg EbSe and 5 mg/kg Ag^+^ and DMSO on the 1-, 3-, and 5-days post-infection, separately.

### Bioluminescent Imaging Analysis

Mice were deprived of food and water for 16 h prior to intragastric administration with YpIII-bioluminescent and were monitored for bioluminescent emission using IVIS lumina II (Caliper LifeSciences) at 1-, 4-, and 7-days post-infection. Mice were anesthetized using the XGI-8 gas anesthesia system (Caliper LifeSciences) prior to imaging with 2.5% IsoFluVet in oxygen (Orion Pharma Abbott Laboratories Ltd., Great Britain), and during imaging in 0.5% IsoFluVet. Images were acquired and analyzed using Living Image 4.5 (Caliper LifeSciences). To analyze bacterial localization within gastrointestinal tracts, mice were euthanized, the gastrointestinal tracts were removed, and imaged by bioluminescent imaging.

### Immunohistochemical Analysis

Mice intestinal tracts were rolled, fixed in formalin (10%), and embedded in paraffin. Paraffin sections (4 μm thick) were used to detect cytokine levels by H&E stain and immunohistochemical (IHC) analysis. Anti-IL-1β, anti-TNF-α and anti-IL-10 antibodies were used for the analysis of cytokines in intestinal tracts.

### Blood Routine Analysis

Mice blood was collected 7 days post-infection, and the white blood cells (WBCs) and neutrophils counts were detected. For investigation of liver and kidney function, blood was collected and centrifuged at 3,000 rpm for 10 min. Serum alanine transaminase (ALT), aspartate aminotransferase (AST), urea and creatinine were determined by fully automatic biochemical analyzer (Siemens, viva proE).

### Statistical Analysis

Statistical analyses were performed by GraphPad Prism 6.0 (GraphPad Software). Means of data between two groups were contrasted using unpaired Student’s *t* test. Sample rates between two groups were tested with chi-square analysis. *p*-values of < 0.05 were significant.

## Results

### EbSe-Ag^+^ Has Strong Bactericidal Activity Against YpIII

**FIGURE 1:**
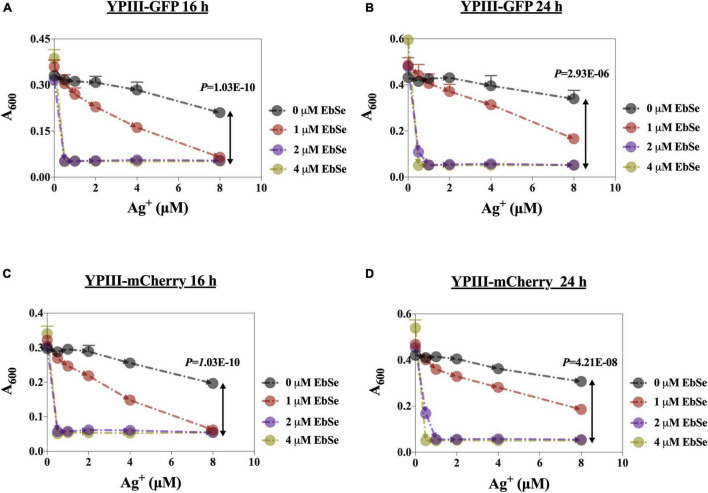
EbSe-Ag^+^ shows synergistic antibacterial activity against YpIII. YpIII-GFP and YpIII-mCherry were cultured to A_600_ of 0.4, diluted 1:1,000 times, and treated with EbSe-Ag^+^. **(A,C)** At 16 h; **(B,D)** at 24 h; **(A,B)** YpIII-GFP; **(C,D)** YpIII-mCherry.

The antibacterial effects of EbSe-Ag^+^ on the growth of YpIII-GFP and YpIII-mCherry were detected by UV-Vis. As shown in Figure 1, Ag^+^ alone inhibited YpIII growth with a 90% minimal inhibition concentration (MIC_90_) of 8 μM, while the addition of 2 μM EbSe effectively reduced the MIC_90_ of Ag^+^ to 0.5 μM (16 times). Meanwhile, 2 μM EbSe and 0.5 μM Ag^+^ showed no synergistic toxicity on human cells as described previously (Zou et al., 2017). In addition, the formula S = (F_X0_/F_00_) * (F_Y0_/F_00_) – (F_XY_/F_00_) was used to calculate the synergistic effect of EbSe-Ag^+^. The results showed that the synergy indexes were 0.97 and 0.94, respectively, indicating EbSe-Ag^+^ has a strong synergistic antibacterial effect against YpIII (Supplementary Figure 1).

**FIGURE 2:**
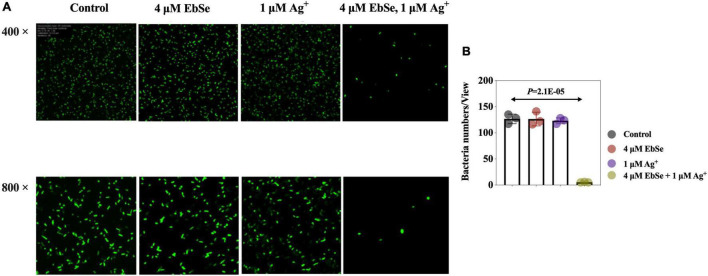
EbSe-Ag^+^ has a bactericidal effect against YpIII. YpIII-GFP were cultured to A_600_ of 0.4, diluted 1:1,000 times, and treated with either EbSe, Ag^+^ or EbSe-Ag^+^. **(A)** LSCM results showed EbSe-Ag^+^ has a bactericidal effect against YPIII-GFP; **(B)** quantitative values.

Whether the antibacterial effect of EbSe-Ag^+^ is bacteriostatic or bactericidal was further investigated by LSCM and bacterial plating. 4 μM EbSe and 1 μM Ag^+^ were inoculated with YpIII-GFP and observed by LSCM (Figure 2). Meanwhile, the minimum bactericidal concentration (MBC) was calculated by plating (Supplementary Figure 2). Both results showed that the combination of 4 μM EbSe and 1 μM Ag^+^ has significant bactericidal effects against YpIII-GFP.

**FIGURE 3:**
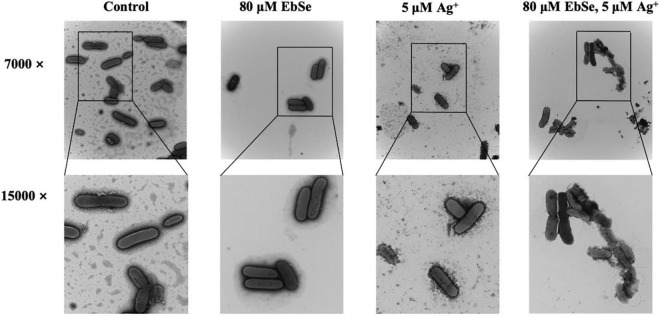
EbSe-Ag^+^ influences YpIII morphology. YpIII-GFP were cultured to A_600_ of 0.4 and treated with EbSe-Ag^+^. TEM results showed EbSe-Ag^+^ influences YPIII-GFP morphology.

Further, the effect of EbSe-Ag^+^ on the morphology of YpIII was assessed by TEM. Normal YpIII-GFP has a smooth surface and a complete cell membrane and cell wall. After 30 min treatment with 80 μM EbSe and 5 μM Ag^+^, YpIII-GFP cells were deformed, cell membranes and cell walls were ruptured, and overflow of cell contents; meanwhile, 80 μM EbSe or 5 μM Ag^+^ treated cells showed no obvious morphological changes (Figure 3). The results showed that the morphology of YpIII-GFP changed significantly in EbSe-Ag^+^ treated group compared with the control group.

### EbSe-Ag^+^ Has Outstanding Post-antibiotic Effect Against YpIII

**FIGURE 4:**
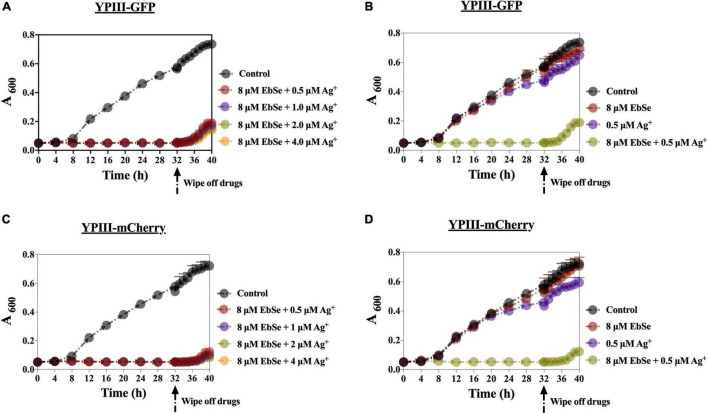
The PAE effect of EbSe-Ag^+^ against YpIII. YpIII-GFP and YpIII-mCherry were cultured to A_600_ of 0.4, diluted 1:1,000 times, and treated with EbSe-Ag^+^. The results showed EbSe-Ag^+^ has a PAE of 4 h. **(A,C)** 8 μM EbSe and a serial concentration of Ag^+^; **(B,D)** 8 μM EbSe and 0.5 μM Ag^+^; **(A,B)** YpIII-GFP; **(C,D)** YpIII-mCherry.

YpIII was cultured and co-cultivated with 8 μM EbSe and 0.5 μM Ag^+^ for 32 h. During this period, the A_600_ was measured every 4 h. 32 h post-treatment, the bacterial solution was centrifuged and washed in PBS by 3 times, and resuspended in LB. The A_600_ was further detected every 1 h for 6 h. The results show that the post-antibiotic effect (PAE) of EbSe-Ag^+^ was 4 h, which is longer than many clinical antibiotics, including quinolone (Figure 4).

### DTT Rescues YpIII From EbSe-Ag^+^ Treatment

**FIGURE 5:**
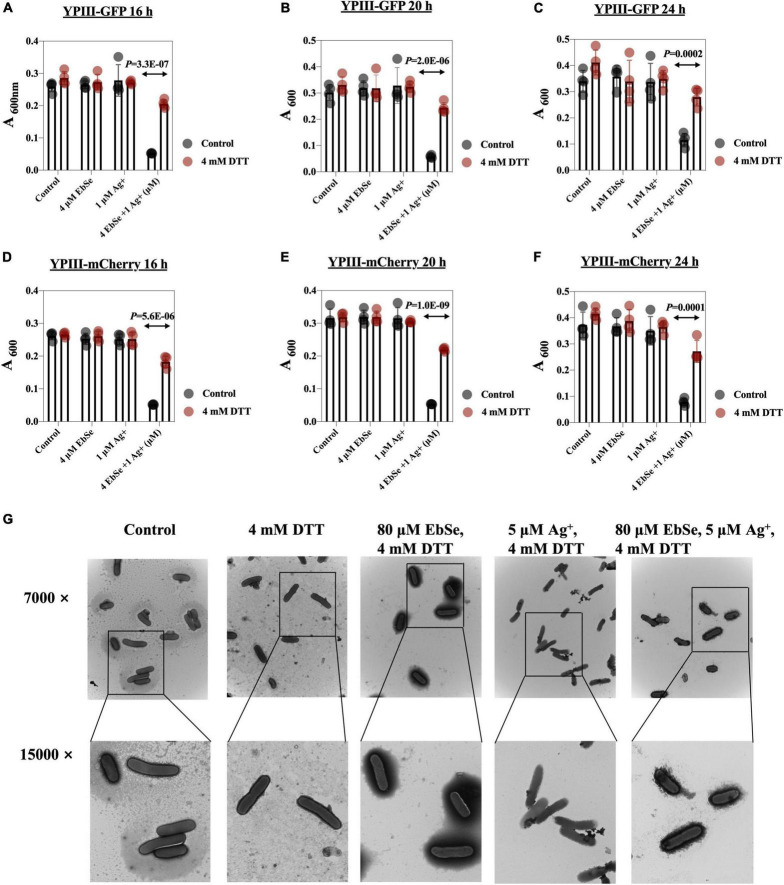
DTT protects YpIII from EbSe-Ag^+^. YpIII-GFP and YpIII-mCherry were cultured to A_600_ of 0.4, diluted 1:1,000 times, and treated with DTT followed with EbSe-Ag^+^. **(A,D)** At 16 h; **(B,E)** at 20 h; **(C,F)** at 24 h; **(A–C)** YpIII-GFP; **(B–E)** YpIII-mCherry. **(G)** YpIII-GFP was cultured to 0.4 and treated with EbSe-Ag^+^ with DTT, and the morphological changes were observed by TEM.

The protective effect of DTT against EbSe-Ag^+^-treated YpIII was evaluated by UV-Vis and TEM assays. DTT was an organic reducing agent that can remove ROS, and 4 mmol/L DTT can effectively rescue YpIII from the bactericidal effect of 4 μM EbSe and 1 μM Ag^+^ (Figures 5A–F). Meanwhile, the TEM results showed that DTT could protect YpIII from EbSe-Ag^+^ caused morphological damage (Figure 5G). Both results indicated that the death of YpIII caused by EbSe-Ag^+^ was related to ROS production.

### EbSe-Ag^+^ Up-Regulates the Intracellular ROS Level in YpIII

**FIGURE 6:**
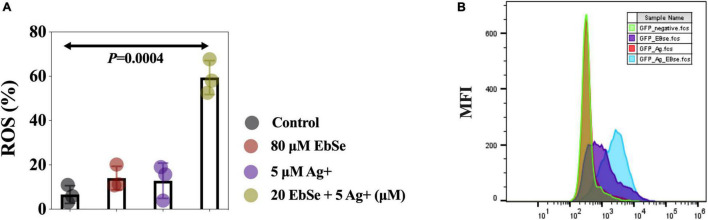
EbSe-Ag^+^ upregulates ROS production in YpIII. YpIII-mCherry was cultured to A_600_ of 0.4 and treated with EbSe-Ag^+^. **(A)** Cells were stained with 5 μM CellROX™ Deep Red Reagent for 30 min at 37°C and detected by FCM; **(B)** quantitative values.

The mean fluorescent intensity (MFI) of ROS in YpIII-mCherry cells was detected by flow cytometry (Ezraty et al., 2017; Zou et al., 2017). The result showed that the ROS production level in YpIII cells treated with 80 μM EbSe and 5 μM Ag^+^ was significantly regulated when compared with the control (Figure 6).

### EbSe-Ag^+^ Disrupts YpIII Thiol-Dependent Redox System

**FIGURE 7:**
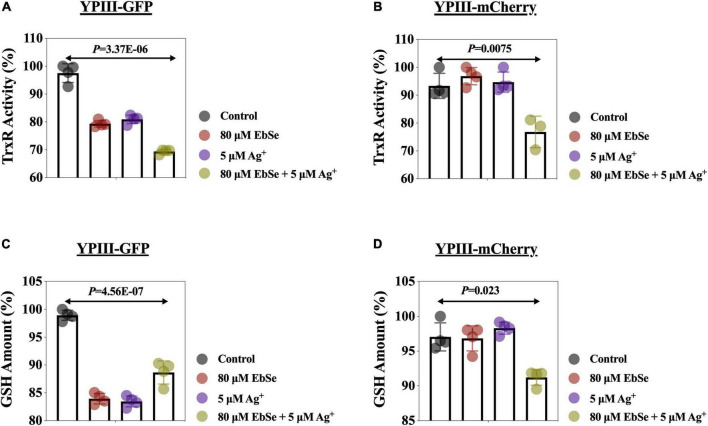
EbSe-Ag^+^ inhibits TrxR activity and depletes GSH in YpIII. YpIII-GFP and YpIII-mCherry were cultured to A_600_ of 0.4 and treated with EbSe-Ag^+^. DTNB assay was used to detect the TrxR activity and GSH amount. **(A,B)** At 30 min; **(C,D)** 0–5 min slope; **(A,C)** YpIII-GFP; **(B,D)** YpIII-mCherry.

The effects of EbSe-Ag^+^ on bacterial TrxR activity and GSH amount in YpIII were measured by DTNB assay. The results showed that the combination of 80 μM EbSe and 5 μM Ag^+^ could efficiently inhibit the TrxR activity and deplete GSH amount when compared with the control (Figures 7A–D).

**FIGURE 8:**
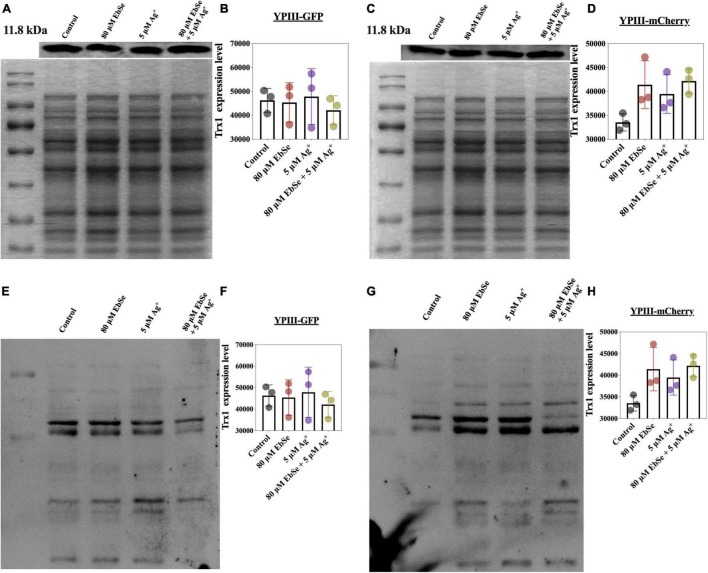
Effect of EbSe-Ag^+^ on Trx1 and *S*-PSSG expressions in YpIII. YpIII-GPF and YpIII-mCherry were cultured to A_600_ of 0.4 and treated with EbSe-Ag^+^. WB assay was used to detect **(A,C)** the Trx1 expression level and **(E,G)** *S*-PSSG expression level; **(B,D)** quantitative values of the Trx1 expression level; **(F,H)** quantitative values of the *S*-PSSG expression level; **(A,B,E,F)** YpIII-GFP; **(C,D,G,H)** YpIII-mCherry.

Whether EbSe-Ag^+^ could affect protein Trx1 and *S*-PSSG expression levels were also analyzed by Western blot (Figures 8A–H). The result showed that Trx1 protein expression level had no difference, indicating the treatment of EbSe-Ag^+^ only influences enzyme activity. Meanwhile, protein *S*-PSSG was reduced by EbSe-Ag^+^ treatment compared with Ag^+^ or EbSe, which further reflected the loss of GSH.

Taken together, the above results showed that EbSe-Ag^+^ inhibited TrxR activity and depleted GSH in YpIII. All the results suggested that EbSe-Ag^+^ could disrupt YpIII TDRS, and ROS production is one of the key mechanisms underlying its bactericidal activity.

### EbSe-Ag^+^ Protects Mice From YpIII-Caused Gastroenteritis

**FIGURE 9:**
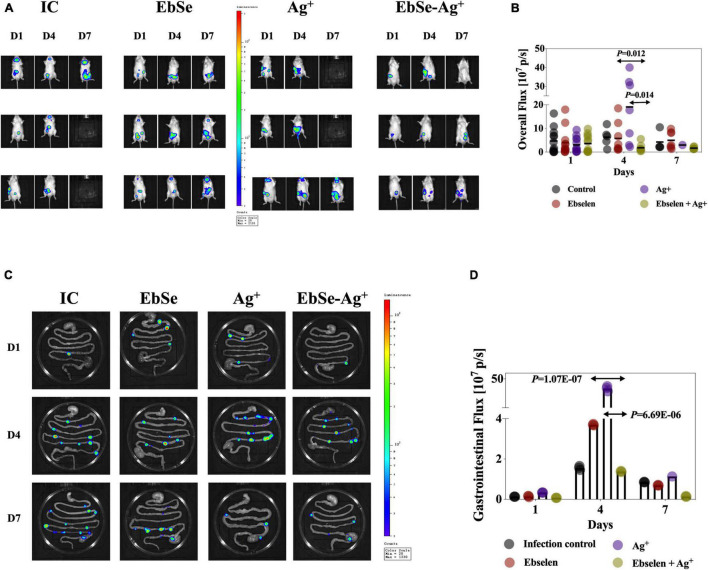
EbSe-Ag^+^ protects mice from YpIII-caused pseudotuberculosis. YpIII-illuminous were orally administrated into mice, and further treated with EbSe-Ag^+^. **(A,B)** Mice were monitored for bioluminescent emission using IVIS lumina II (Caliper LifeSciences) at 1-, 4-, and 7-days post-infection. **(C,D)** Mice were euthanized, the gastrointestinal tracts were removed, and imaged by bioluminescent imaging to analyze bacterial localization within gastrointestinal tracts at 1-, 4-, and 7-days post-infection. IC: infection (illuminous) control.

Gastroenteritis is among the most common *Y. pseudotuberculosis*-caused infections (Amphlett, 2016). To evaluate whether EbSe-Ag^+^ could protect mice from YpIII-caused gastroenteritis, 60 mice were randomly divided into five groups, and 2 × 10^9^ CFU/100 μL bioluminescent-YpIII strain Xen4 (Wang et al., 2016) were orally administrated to construct gastroenteritis models. Mice were further injected i.p. with 20 mg/kg EbSe, 5 mg/kg Ag^+^, 20 mg/kg EbSe and 5 mg/kg Ag^+^ and DMSO on days 1-, 3-, 5-, post-infection. Mice were monitored for bioluminescent emission at 1-, 4-, and 7-days post-infection using IVIS. To further analyze bacterial localization within gastrointestinal tracts, mice were euthanized, the gastrointestinal tracts were removed, and imaged by bioluminescent imaging. The results showed that EbSe-Ag^+^ led to a significant reduction in bacterial load on-site compared with the control group (Figure 9). These findings demonstrated that EbSe-Ag^+^ could effectively protect mice from YpIII-caused gastroenteritis.

### EbSe-Ag^+^ Reduces Inflammatory Responses in Gastroenteritis Mice

**FIGURE 10:**
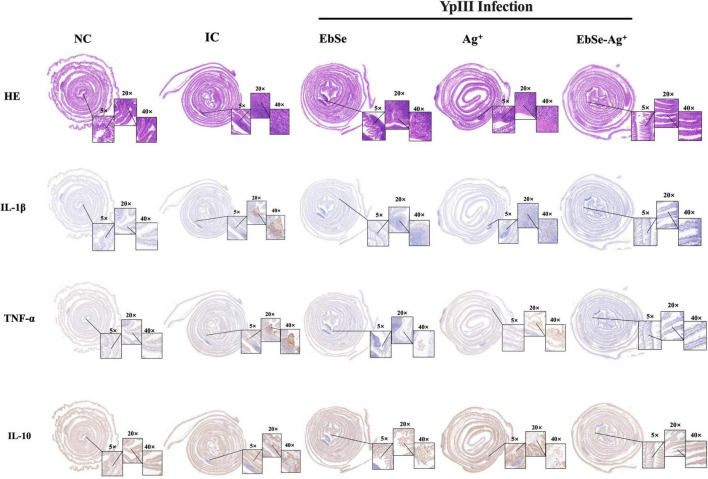
EbSe-Ag^+^ has immune-modulatory property. YpIII-illuminous were orally administrated into mice, and further treated with EbSe-Ag^+^. Mice intestinal tracts were rolled, fixed, and embedded. Paraffin sections were used to perform H&E stain and detect cytokine (IL-1β, TNF-α and IL-10) levels by IHC analysis.

Hematoxylin and eosin (H&E) staining was performed using the new formative tissues. Staining revealed that control mice had increased number of inflammatory cells. Meanwhile, EbSe-Ag^+^-treated mice had less scattered lymphocytes (Figure 10).

An IHC assay was used to measure the presence of pro-inflammatory cytokines IL-1β and TNF-α and anti-inflammatory cytokine IL-10. As shown in Figure 10, EbSe-Ag^+^ could significantly down-regulate IL-1β and TNF-α and up-regulate IL-10 when compared to control mice.

**FIGURE 11:**
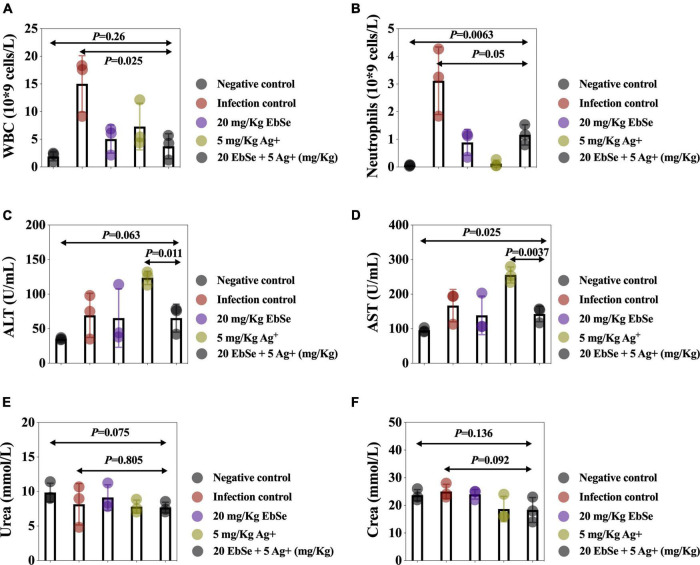
Routine blood analysis of EbSe-Ag^+^-treated mice. YpIII-illuminous were orally administrated into mice and further treated with EbSe-Ag^+^. Mice blood was collected 7 days post-infection, and the numbers of **(A)** WBC and **(B)** neutrophils, the amounts of **(C)** ALT, **(D)** AST, **(E)** Urea, and **(F)** creatine were detected.

The peripheral blood from different groups of mice was collected, and the results showed that EbSe-Ag^+^ statistically reduced the counts of WBCs (Figure 11A) and neutrophils (Figure 11B) compared with the control. Furthermore, ALT, AST, urea, and creatinine in mice blood serum were also detected, and the results showed that EbSe-Ag^+^ treatment reduced the ALT (Figure 11C), AST (Figure 11D) levels compared with Ag^+^ treatment.

Overall speaking, EbSe-Ag^+^ treatment exhibited significant effects in reducing the YpIII-caused inflammation with low toxicity.

## Discussion

The study of the Gram-negative zoonotic bacteria *Y. pseudotuberculosis* has been at the forefront of cellular and molecular pathogenesis for over four decades (Davidson and Davis, 2020). Since *Y. pseudotuberculosis* has a very diverse host spectrum, it can infect virtually all warm-blooded animals, including humans, livestock, pets, and wild animals (Hashimoto et al., 2021). Therefore, given the growing fascination of human populations with wildlife (such as game meat), the pseudotuberculosis can be expected to increase. Here, we showed that EbSe work synergistically with Ag^+^ to eliminate *Y. pseudotuberculosis* YpIII strain *in vitro*. Briefly, UV-vis and LSCM assays verified that the pharmacological synergy was generated; TEM results revealed that EbSe-Ag^+^ caused the deformation, shrinkage, and content overflow of YpIII cells, suggesting the rupture and decomposition of the bacterial cell membrane. Further, DTT was used as organic reductant, and the UV-vis, TEM, and FCM results proved that DTT can protect YpIII from the bactericidal effect of EbSe-Ag^+^, indicating the antibacterial mechanism of EbSe-Ag^+^ is highly related to the overmuch accumulation of ROS.

**FIGURE 12:**
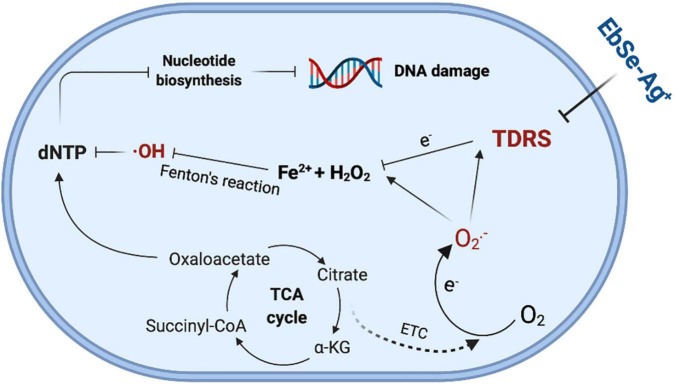
The illustration of bactericidal effect of EbSe-Ag^+^.

Known as a highly reactive oxygen-containing molecule, ROS is continuously produced by mitochondrial metabolism and eliminated by dedicated antioxidant systems (Rabinovitch et al., 2017). Since the excessive ROS oxidize proteins, lipids, and DNA (Lei et al., 2018), its intrinsic characteristics is the basis of the mechanism necessary for the survival and growth of organisms (Li et al., 2017), and the its homeostasis in bacteria plays a crucial role in regulating diverse physiological functions in bacteria (Zhang et al., 2017). Thus, the DTNB assays were performed, and the results showed that the activity of TrxR and the content of GSH were both significantly decreased by EbSe-Ag^+^, indicating the disrupt of redox homeostasis. Furthermore, the expression levels of Trx1 and *S*-PSSG were detected by Western blot as described previously (Zou et al., 2017). The results showed that the expression level of Trx1 maintained, while the level of *S*-PSSG decreased significantly after EbSe-Ag^+^ treatment. Collectively, our results indicated that EbSe-Ag^+^ affected the activity of the enzymes in Trx system rather than its expression; meanwhile, GSH system was significantly disrupted by consumption the GSH pool (Figure 12).

Using well-established experimental gastroenteritis in mice offers the opportunity to evaluate the preventive strategies for pseudotuberculosis (Wang et al., 2016). In the current study, the infected mice were treated by intraperitoneal administration of EbSe-Ag^+^. The mice were monitored for bioluminescent emission at regular intervals, and the therapeutic effect of EbSe-Ag^+^ was verified by a significant reduction in bacterial loads (Avican et al., 2017; Brodl et al., 2018; Fleiss and Sarkisyan, 2019). Pro-inflammatory cytokines, TNF-α, and IL-1β, which are produced during intestinal inflammation have important intestinal epithelial tight junction barrier-modulating actions (Kaminsky et al., 2021), highly related to inflammatory diseases of the gut (Xu et al., 2018). Our results showed that both TNF-α, and IL-1β were down-regulated by EbSe-Ag^+^, contributing to host defense during infection. Given that IL-10 is at the center of maintaining the delicate balance between effective immunity and tissue protection, it is not surprising that IL-10 is particularly important in maintaining the intestinal microbe-immune homeostasis, and functions to prevent excessive inflammation during the course of infection (Neumann et al., 2019). Our results showed that EbSe-Ag^+^ up-regulated IL-10 that provide a favorable outcome in host healing, which might be highly related to its recognized immune-modulatory, anti-inflammatory, and antioxidant activities (Ren et al., 2018). Neutrophils are critical components of immunity and can rapidly migrate to infected or injured tissue, performing central roles in both the innate and the acquired immune systems (Lemaitre et al., 2022). Upon their activation, release of signaling molecules by neutrophils help combat bacterial pathogens (Borregaard, 2010; Wang L. et al., 2020). The enteropathogenic bacterium YpIII successfully persists as an extracellular bacterium during infection by virtue of its translocation of virulence effectors that act in the cytosol of host immune cells to subvert phagocytosis and proinflammatory responses (Taheri et al., 2016). In our animal trial, the peripheral blood of mice was collected, and the neutrophil counts were detected. The result showed that EbSe-Ag^+^ can reduce the production of inflammatory cells. In addition, ALT and AST were monitored, and the results showed that the enzymes in the EbSe-Ag^+^ group were significantly lower than that of in the Ag^+^ group, proving that the EbSe-Ag^+^ could reduce the hepatotoxicity caused by Ag^+^ to a certain extent. In parallel, blood urea and serum creatinine were also monitored. The results illustrated that EbSe-Ag^+^ in this experiment has no obvious nephrotoxicity in mice.

## Conclusion

Our *in vitro* study demonstrated that EbSe work synergistically with Ag^+^ to eliminate YpIII by disrupting TDRS; meanwhile, the *in vivo* experiment verified the antibacterial activity and immune-modulatory property of EbSe-Ag^+^. Taken together, all the results help to develop its scientific basis for pseudotuberculosis control.

## Data Availability Statement

The original contributions presented in the study are included in the article/Supplementary Material, further inquiries can be directed to the corresponding author/s.

## Ethics Statement

The animal study was reviewed and approved by the ethical permit approval of the Medical Animal Care and Welfare Committee of China Three Gorges University (2019080Q) was obtained before using the mice for study.

## Author Contributions

CD, WC, and PW performed *in vitro* experiments. JW and HW analyzed and interpreted the data. BL, KD, DG, and HC performed animal experiments. LZ was a major contributor in writing the manuscript. All authors read and approved the final manuscript.

## Conflict of Interest

The authors declare that the research was conducted in the absence of any commercial or financial relationships that could be construed as a potential conflict of interest.

## Publisher’s Note

All claims expressed in this article are solely those of the authors and do not necessarily represent those of their affiliated organizations, or those of the publisher, the editors and the reviewers. Any product that may be evaluated in this article, or claim that may be made by its manufacturer, is not guaranteed or endorsed by the publisher.

## Supplementary Material

Supplementary Figure 1 The S degree of EbSe-Ag+ against YpIII. UV-Vis assay was used to measure the survival of bacteria and the result showed that the S degree of 4 μM EbSe and 0.5 μM Ag^+^ was 0.97 and 0.94, respectively (in this figure). S = (F_*X0*_/F_00_) * (F_*Y0*_/F_00_) - (F_*XY*_/F_00_).

Supplementary Figure 2 EbSe-Ag+ showed bactericidal activity against YpIII. YpIII-GFP and YpIII-mCherry were cultured to A_600_ of 0.4, diluted 1:1,000 times, treated with EbSe-Ag^+^ and plated on LB medium.
