# Supplementary figures and images for: The Assessment on Synergistic Activity of Ebselen and Silver Ion Against Yersinia pseudotuberculosis
Source: Front Microbiol. 2022 Jul 25;13:963901. doi: 10.3389/fmicb.2022.963901 (PMC9363147; doi:10.3389/fmicb.2022.963901)

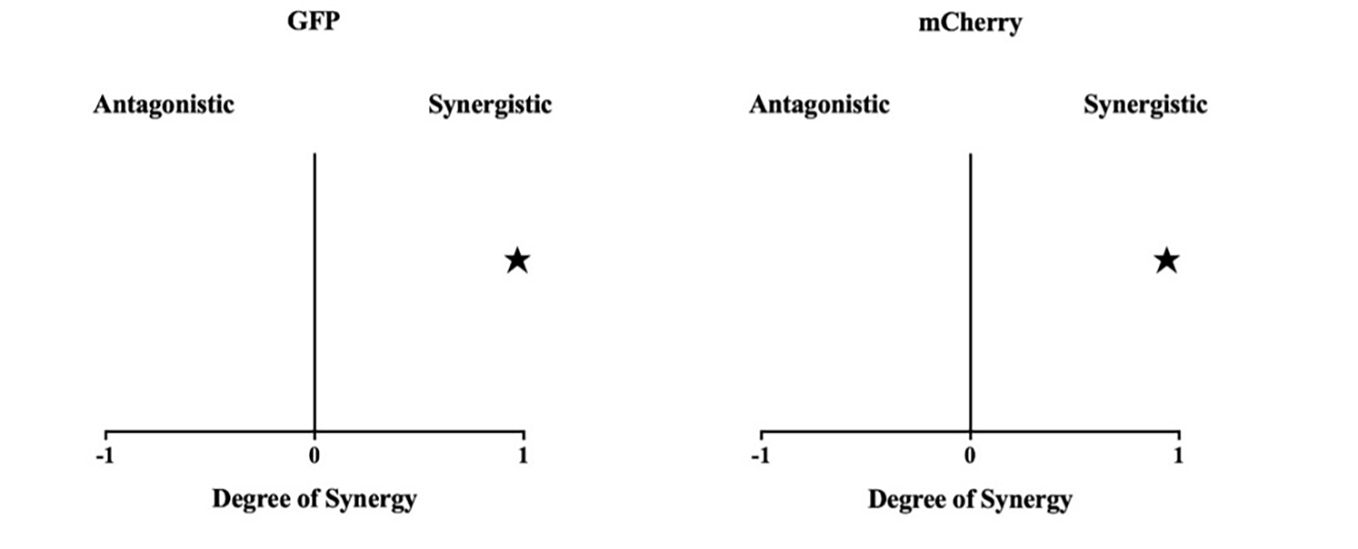

Supplement: Supplementary Figure 1 — The S degree of EbSe-Ag+ against YpIII. UV-Vis assay was used to measure the survival of bacteria and the result showed that the S degree of 4 μM EbSe and 0.5 μM Ag+ was 0.97 and 0.94, respectively (in this figure). S = (FX0/F00) * (FY0/F00) - (FXY/F00). [file Image_1.JPEG]

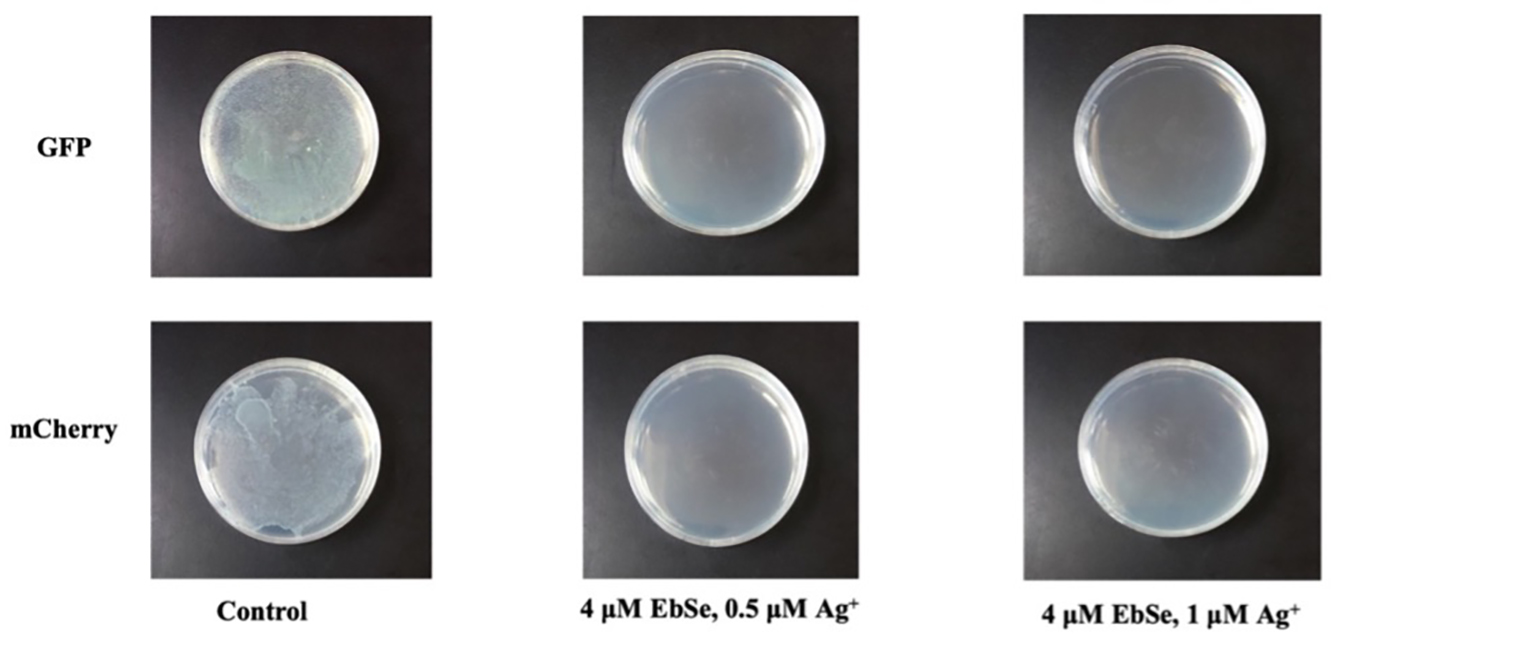

Supplement: Supplementary Figure 2 — EbSe-Ag+ showed bactericidal activity against YpIII. YpIII-GFP and YpIII-mCherry were cultured to A600 of 0.4, diluted 1:1,000 times, treated with EbSe-Ag+ and plated on LB medium. [file Image_2.JPEG]
